# Assessing the performance of QSP models: biology as the driver for validation

**DOI:** 10.1007/s10928-023-09871-x

**Published:** 2023-06-29

**Authors:** Fulya Akpinar Singh, Nasrin Afzal, Shepard J. Smithline, Craig J. Thalhauser

**Affiliations:** Genmab US, Inc., 777 Scudders Mill Rd Bldg 2 4th Floor, Plainsboro, NJ 08536 USA

**Keywords:** Quantitative systems pharmacology, Model validation, Model assessment, Model calibration

## Abstract

Validation of a quantitative model is a critical step in establishing confidence in the model’s suitability for whatever analysis it was designed. While processes for validation are well-established in the statistical sciences, the field of quantitative systems pharmacology (QSP) has taken a more piecemeal approach to defining and demonstrating validation. Although classical statistical methods can be used in a QSP context, proper validation of a mechanistic systems model requires a more nuanced approach to what precisely is being validated, and what role said validation plays in the larger context of the analysis. In this review, we summarize current thoughts of QSP validation in the scientific community, contrast the aims of statistical validation from several contexts (including inference, pharmacometrics analysis, and machine learning) with the challenges faced in QSP analysis, and use examples from published QSP models to define different stages or levels of validation, any of which may be sufficient depending on the context at hand.

## Introduction

### Preliminaries

The efficient discovery and development of a safe and efficacious drug requires a mechanistic understanding of physiological and biochemical processes that contribute to drug exposure and efficacy. Quantitative systems pharmacology (QSP) modeling is a modeling and simulation approach to integrate the data and knowledge mechanistically and quantitatively, which allows not only representation of existing data, but also extrapolation of current knowledge to untested scenarios. The predictive capability of QSP models is crucial for hypothesis generation to support target selection, optimization of dosing strategy, biomarker identification, patient stratification and combination strategies.

QSP model development starts with establishing the model structure and parameter ranges by integrating a multitude of qualitative and quantitative data relevant to biological system and disease of interest, such as in vitro and in vivo experiments, transcriptomics and proteomics data and clinical data. Due to complexity of the biological systems QSP models tend to have many parameters. While aim is to constrain these parameters by direct measurements, often majority of the parameters are not measurable, or laboratory measurements do not translate to in vivo situation. Therefore, the techniques such as sensitivity analysis and model reduction are important to identify the most impactful parameters and to reduce the problem size in the next stage of model development when the modeler navigates through the uncertainties in the predictions due to nonlinearity and nonidentifiable parameters that are unique to large models like QSP models.

As with other pharmacometrics modeling approaches, a distinctive feature of QSP models is their ability to represent interpatient variability. With large set of sensitive parameters, a plethora of outcomes can be simulated, and thereby, tweezing the variability from uncertainty and elucidating the cause of the observed variability becomes a challenging problem. To address this, QSP modelers employ virtual population (VPop) techniques, where a unique set of parameters are used to simulate an individual virtual patient (VP) and interpatient variability is represented in VPop by varying these parameters. These virtual populations allow for the mechanistic exploration of how observed variability in a population might be formed (e.g., how intrinsic production capacity, cell numbers, and cell activation potential can all contribute to plasma cytokine concentration). There may be many ways to generate a certain range or distribution of any single observable. To capture observed range of responses and interpatient variability accurately and to increase the predictive capability of the model the VPop needs to be calibrated and validated across the spectrum of biology supported in the model.

### What is calibration and validation in QSP?

The aim of the calibration in QSP is to capture the distribution of observed responses in a population-level data, such as clinical study data. Generally, the process begins by simulating a large cohort of biologically plausible VPs, which all meet certain requirements, but which do not accurately represent the calibration data. From this cohort, one could follow a number of strategies. Firstly, one could algorithmically select a sub-population of these VPs to become the final VPop, so that these VPs together match the calibration data. In this case, all VPs contribute equal information as to the population predictions. Alternatively, one could construct a means to sample the larger cohort such that on average, samples of VPs reproduce the calibration data. In this case, the information content of each VP is proportional to its probability of being selected [1, 2]. This approach allows for the creation of populations of differing sizes and explorations of questions of power analysis.

While calibration strengthen the ability of the QSP model to capture the available data, validation can test the ability of the QSP model to extrapolate to untested scenarios by testing how the distribution of responses from calibrated VPop compares with observed responses from studies that were not used in calibration. Since QSP model validation depends on the VPop generated in calibration step, validation in QSP cannot be considered in isolation and can be seen as a complementary component of calibration, both strengthening the predictive power of QSP models [3–5]. This is also true for QSP models that do not employ VPop approach, as the data used in validation is tied to data used in calibration [6].

## Approaches to validation in QSP

### Approach to validation in QSP is context-dependent

QSP modeling can be used to describe many different biological systems and address a diverse range of questions. Therefore, approaches and rigor in validation of QSP models can vary depending on the scope and the availability and the nature of the data [5, 7, 8]. For instance, the models that aim to predict clinical outcomes need to be calibrated and validated with clinical data. If the clinical data provides sufficient insights on individual patient responses and their distributions VPop approach can be utilized [1, 2, 9–11]. On the other hand, if a model is developed in an area where only some preclinical data is available a rigorous approach may not be possible. In this case a qualitative assessment of the model behavior may be sufficient if model is aimed to address exploratory or preclinical questions. As it may be apparent in this second example, the data availability can also impact the scope of the model and therefore, can shape the entire QSP model development process. Therefore, it is important to understand the type of data and the way the data is utilized in the QSP model development.

### What is data used in the QSP model development?

Development of QSP models typically evolves with the generation of the new data and information in the related disease area. In vitro measurements and qualitative behaviors observed in preclinical and clinical studies can inform the model structure and initial model parameterization. If direct measurement is not possible, parameter estimation by fitting a mini-model (a separate model to capture a specific experiment) to the related in vitro data is a common technique [6, 9]. The model parameters can be further fine-tuned by fitting the unknown model parameters to the mean of related observations from in vivo experiments or clinical studies [12, 6, 13–17]. For instance, Riggs et al. fit their model to data compiled from five different clinical studies to capture the average patient behavior with regards to relationship between estrogen and bone mineral density and impact of intervention for management of endometriosis [13]. In this work and in a follow-up work [18] the modelers validated the model based on the mean behavior as it was sufficient to address the questions of interest. In another example, Kosinsky et al. employs NLME to estimate the model parameters and variability simultaneously using a relatively rich in vivo tumor growth data set [17]. This process of parameter fine-tuning can be referred as model calibration by some modelers and should not be conflated with VPop calibration [12, 6].

In relatively more data-rich areas, we see more QSP models that are further calibrated with VPop approach [9, 10, 2, 19, 20, 1]. Especially patient-level data enables the modelers to test the plausibility and the prevalence of the patient response phenotypes simulated by the QSP models. Kirouac et al. is a good example of how a modeler can embrace aforementioned parameter estimation technique along with VPop approach to integrate a wealth of data from preclinical and clinical studies [9]. In this work, the authors calibrated the VPop by estimating relative prevalence of tumor growth phenotypes from their QSP model describing the link between EGFR activation, MAPK signaling pathway and tumor growth using clinical data from three Ph1b studies with combinations of EGFR, BRAF and MEK inhibitors. The model was later validated with another clinical study with an ERK inhibitor, a drug on the same pathway.

Kirouac’s approach to partitioning the data sets for VPop calibration and validation is not uncommon. Many other QSP studies that either validate only the mean model predictions or validate both mean and interpatient variability via VPop approach use data of the drugs on the same pathway, but support calibration and validation processes with different clinical studies or drugs [13, 20, 19, 21]. For instance, Riggs et al. assigned data from 5 studies with GnRH agonist or antagonist treatment to calibration set and 9 studies to validation set [13], and later the model was further calibrated with 4 Ph3 studies [18]. Similarly, Gadkar et al. used Atorvastatin Ph2 study and anti-PCSK9 SAD study for calibration step, and anti-PCSK9 MAD study and a Ph2 study with combination of Atorvastatin and anti-PCSK9 for validation [19]. Alternatively, data can be partitioned based on dose regimen of the same drug [22], based on biomarkers from the same studies [10], based on species [6, 15], or based on study type (i.e. observational studies for calibration and intervention studies for validation) [23].

### Biology as the driver for validation

Before considering how one might approach validating a QSP model, the question must first be asked what precisely is being validated in the model. Structure and parameterization of components of the model might be evaluated in the context of in vitro or preclinical experiments, but in the clinical context the model is used to generate a VPop that capture the observed variability in biological state and outcome. Modelers are faced with design choices about how much biology to include in the model. One might settle upon a large scope of biology, even if there is no clinical data to constrain the pathway activities in the clinical population, if there is sufficient molecular data to be reasonably certain as to its parameterizations and contributions in the disease state. On the other hand, one may adopt a more parsimonious approach, in which only the biology needed to represent the desired clinical intervention(s) is considered, with other biology involved in the disease subsumed into lumped parameters and processes. The first strategy allows one to explore therapy combinations and molecular causes of response (or non-response) at the expense of significantly more parameters involved in the VPop generation and calibration process, with the potential for one or more of the pathways involved in generation being unconstrained by the calibration data. The second strategy allows for more focused exploration of variability in the clinical context; however, opportunities to mechanistically extrapolate are much more limited. In either case, the question must be formulated to probe the VPop for which components are not constrained by the available data, and how those pathways might contribute to the predictive power of the model and its VPop.

### Levels of validation in QSP

With the principal of “biology as driver for validation” in mind, we can categorize QSP model validation approaches in four levels: qualification, within-target validation, within-pathway validation, and cross-pathway validation. Qualification-level validation tests whether the model can generate the intended model outcomes, e.g. observed qualitative behavior of a biomarker or an outcome that directly depends on a marker that is used in calibration. Qualification confirms the ability of the model to interpolate within the observed range of outcomes and increase confidence in model predictions for the tested therapy. Many preclinical and some early-phase clinical QSP models rely on this approach either because the data is limited [6, 15, 16, 21, 23] or because interpolation of the data is sufficient for the scope of the model [12]. In another case, all the clinical data was used to construct VPops so that the subsequent analyses could be well-qualified [2].

**Fig. 1:**
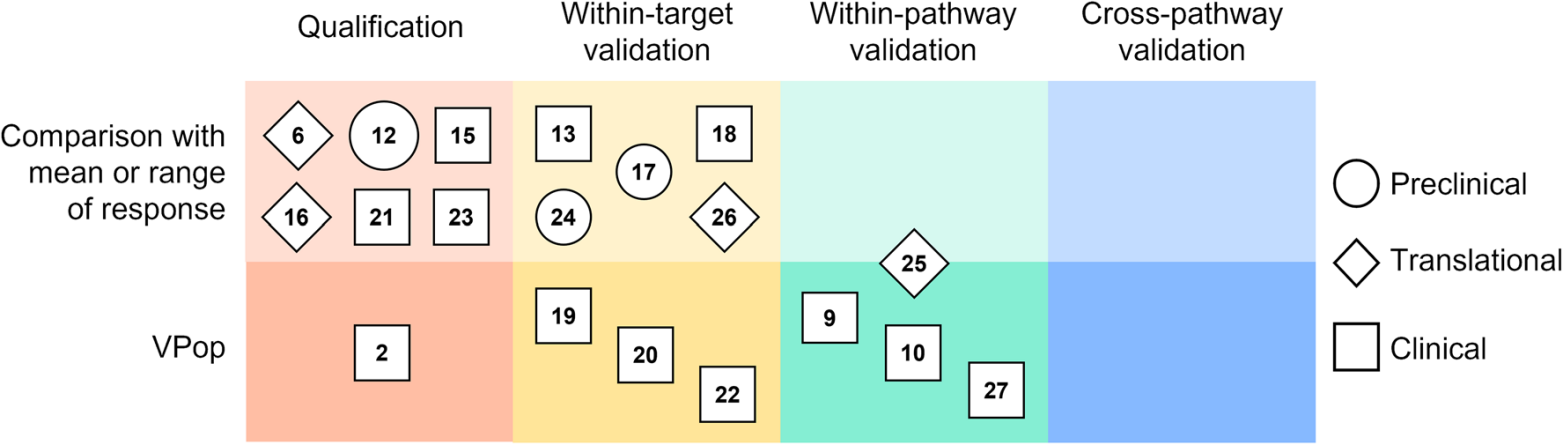
This visual table groups examples from QSP validation literature by the highest level of validation (qualification, within-target validation, within-pathway validation and cross-pathway validation) and calibration/validation technique (comparison with mean or range of response vs. VPop). The numbers indicate the reference number of the published QSP modeling work, while the shape indicates the scope of modeling work

In the therapeutic areas with more abundant clinical data, we start seeing examples of within-target and within-pathway validation. Within-target validation uses the data from a therapy that has the identical mechanism of action as the therapy used for calibration or the data from a different clinical trial with the same drug or drug combination [13, 17–20, 22, 24–26]. To be clear, we are defining target as the specific component of a mechanism or pathway that is subject to experimental perturbation (i.e. the target of the pharmacological intervention). Taking this approach to the next level, some QSP studies also utilize data from other targets within the pathway for validation, which we referred as within-pathway validation [9, 10, 27]. Most of the clinical QSP modeling studies fall under these two categories, and many of these studies use VPop technique as the model complexity increases (Fig. 1).

While the first three levels of validation cover the approaches in the published QSP validation studies, here we would like to introduce a fourth level of validation: cross-pathway validation. As the name indicates in this case, the QSP model is validated with clinical data of therapies with a mechanism of action that is different from, but related to the therapy that the model was calibrated with. This final approach requires significant amount of clinical data as well as extra time investment in model validation on top of the QSP model development and calibration, making it hard to achieve within the typical drug development timeline. However, applying cross-pathway level validation can increase the predictive capability of QSP models significantly especially in the areas where combination therapies targeting different pathways. If the model is validated by perturbing a pathway not included in its primary calibration data set and recapitulates accompanying data for that perturbation, it demonstrates that not only are individual pathways well-constrained (either by data used in the full model calibration or during construction of the individual pathways or model subcomponents) but also so are the various mechanisms by which these pathways interact with one another. This can in turn build confidence in future model predictions, such as the presence (or lack) of synergy in a novel combination regimen.

**Fig. 2:**
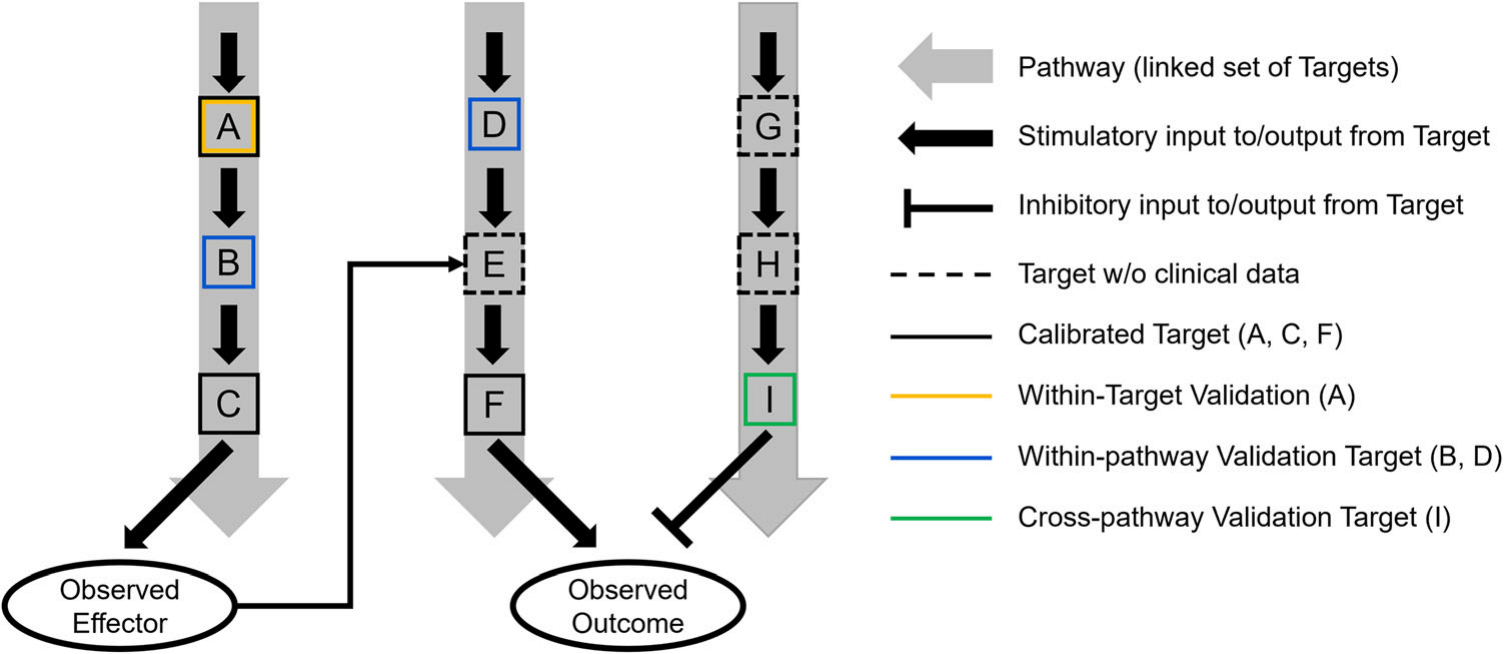
This figure depicts a hypothetical QSP model and its calibration/validation scheme at a given stage of model development. QSP model encompasses a biological system with various biological entities, such as targets and biomarkers (gray block arrows) and indirect relationships (black lines between gray arrows). In this hypothetical calibration/validation scheme, the biological entities in the central pathway are calibrated with preclinical or clinical data (black boxes), while some biological entities are not calibrated (dashed boxes). This hypothetical QSP model is also validated using multiple levels of validation. Some biological entities and their interactions within the central pathway are validated by within-target validation (yellow box) and within-pathway validation (blue box). Data on the entities that are linked to both central pathway and other related pathways are used for cross-pathway validation (green box) (Color figure online)

To illustrate the differentiation between within target, within pathway, and cross-pathway validation, consider a biological model consisting of three pathways as shown in Fig. 2: pathway 1 (components A, B, and C); pathway 2 (components D, E, and F); and pathway 3 (components G, H, and I). All pathways and components therein are well-established in the literature with in vitro data sufficient to parameterize their effects. The output of pathway 1 is a biomarker which plays a regulatory role in pathway 2. Pathways 2 and 3 work in competing manners to influence an observable outcome in a patient. Pharmacological manipulation of pathway 1 (component A, two distinct pharmaceutical agents; component B, one agent, and component C, one agent) and pathway 2 (component D, one agent; component F, one agent) is available, and a virtual population is calibrated using intervention and response (both observed effector and observed outcome) data for components A, C, and F. The remaining data now permits all three levels of validation that has been discussed. A within target validation can be performed by using the second agent influencing component A. A within pathway validation can be performed using data from the agents affecting components B (pathway 1) and D (pathway 2), since the role of these pathways has already been constrained by the calibration data. Finally, based on the calibration data pathway 3 has not been fully constrained; its effects are inferred based on the calibration of pathway 1 (negative regulator of pathway 2) and pathway 2 (enhancer of the observed outcome). A cross-pathway validation using intervention data against component I allows one to demonstrate the validity of the inferred magnitude of pathway 3’s contribution to the observed outcome. Overall, such multi-level validation improves the prediction capability of the model for the validated targets and biomarkers, and also expands the scope of the prediction capability of the model.

The data and modeling approach in Schmidt et al. provides a nice example of rich dataset and QSP model framework that is suitable for application of multiple levels of validation, including cross-pathway validation [2]. In this work, data from 3 classes of therapeutics [anti-TNF (two distinct compounds), anti-IL6R (two doses), and anti-CD20 (B cell depletion)] were utilized. While the authors chose to use the entirety of the data for model calibration, the data could also be partitioned between calibration and validation. For instance, within-target validation can be approached by withholding one dose level of the anti-IL6R and/or one of the two anti-TNF agents. Moreover, cross-pathway validation can in principle be achieved by withholding an entire class of therapy from the calibration process. Permutations of this process are also possible, including a kind of cross-pathway validation scheme in which distinct VPops were calibrated to any two of the three therapies and predicted the third. This type of approach can yield insight into which therapeutic mechanisms provide more (or less) information as to the expected response to another. It can also demonstrate that all the data was necessary to constrain the many degrees of freedom the model had to generate plausible virtual patients, reinforcing the critical need for varied, high-quality data in the QSP development process.

### How to show a QSP model validation?

Some common validation techniques in more traditional pharmacometrics and statistical models can be applied in validation of QSP models. For instance, visual predictive checks (VPCs) are commonly used to assess the ability of a structural NLME model and its estimated parameters (for both fixed and random effects) to properly describe both the central tendencies and the variability of the observed data. Similar procedures have been described for mechanistic models [28, 29]. These tools sample model parameters either around their precision estimates calculated during estimation [29] or by taking as input an explicit random effect [28] and generate Monte Carlo simulations, plotted in manners analogous to the NLME VPC. While these routines are conceptually very similar to VPop strategies, they have been applied to reduced models (as part of model selection) or more traditional pharmacometric models, and to date the literature does not have examples of such tools applied to platform or disease-level QSP models.

When considering the quality of parameter estimates and the predictability of a model, goodness of fit (GoF) metrics can be applied to provide confidence in the model simulations. In the current QSP literature, many of these metrics are qualitative in nature, allowing the reader to visually inspect what features of the data the model describes well, which aspects of the simulation fall short of the data, and whether such gaps between model and data are meaningful. Models described in [30, 31] demonstrate the value of iterative “unit checks” in the model building and validation process, allowing biology and data of differing nature and scope to be layered in to the overall model while preserving past results. More quantitative measures of GoF have been described in the literature, albeit more in the context of calibration rather than validation [32].

The VPop approach for QSP adds further complexity to the question of validation. This approach allows a modeler to use the QSP model in a meta-analysis context, in which published population-level clinical data for different therapeutic strategies are all simulated in the same VPop. In this case, a VPC approach would not be warranted. Instead, with a VPop that can be sampled, one could perform an analysis akin to a bootstrapping, in which populations of defined size are sampled, and used to construct a prediction interval for the model’s ability to reproduce the population-level data [32]. The range of VPop predictions (i.e. the prediction interval for the VPop) can also be simply compared to the observed range of data used in a validation set, such as the within-pathway validation shown in [25].

### How does validation in QSP differ from validation in statistical models

In statistical applications, it is common to discuss validation of the model as being internal or external in nature. Naively, the distinction is made based on the origin of the data used to compare with model predictions: if the source is from the same experiment(s) from which the model parameters were estimated, but withheld from the estimation itself, it would be termed internal validation. Conversely, if the data is derived from a distinct set of experiments, it would be considered an external validation. As stated previously, the objective of these validation steps is to characterize the quality of the parameter estimates and assess the bias and degree of overfitting the model might contain. In this section, we summarize what validation means in these other contexts, to reinforce the scientific and technical differences between these approaches.

### Validation in NLME based pharmacometric modeling and statistical/deep learning

NLME based phamacometric modelling and Statistical/Deep Learning constitute a broad range of algorithms that seek to uncover patterns from large scale and often heterogeneous data sets to make predictions based on these patterns. Pharmacometric modeling is a special case of statistical learning that employs a nonlinear mixed-effect modeling (NLME) strategy and is used in essentially every drug development program. This approach seeks to characterize the total variability of an observable in a population (e.g., the pharmacokinetics of a compound) and to quantify how known input variability in the population (body size, age, gender, renal function, CYP enzyme expression) informs the output variability, so that ultimately one can predict how changes in a clinical experiment (protocol, alternate patient population with defined characteristics) would lead to changes in the observed outcome.

In pharmacokinetic modeling, and more generally, in statistical learning models, training is typically an exercise in parameter estimation. Techniques to estimate parameter and model credible intervals are well-established. These techniques emphasize visual analysis of the assumptions of the model, such as distribution of random effects, correlations between effects and covariates, predicted distributions of model predictions.

If we have sufficient data, these models are best validated by dividing the data into three groups: a training set to train the model, a validation set from which we select the model that gives the smallest prediction error and a test set that measures the overall performance of the model. The goal of splitting the data into train, validate and test sets is to reduce model variance, the sensitivity of the model to minor changes in the training data, while simultaneously minimizing model bias, the error introduced by approximating the complex process being investigated with a simpler model. Finding this balance is guided by the Bias-Variance Decomposition theorem [33].

In situations where there is not enough data to split into three groups researchers typically use k-fold cross-validation. This technique results in models whose test error rates have neither high variance nor high bias, consistent with the Bias-Variance Decomposition theorem [33]. Cross-validation, though more challenging to apply to NLME models due to possible changes in the distribution of random effects, is still useful for structural and covariate model selection [34].

Although typically models are trained, validated, then tested on external data sets prior to publishing the model, there are examples in the literature of external validation performed on population pharmacometrics models after the fact. In separate studies focused on vancomycin [35], meropenem [36], and phenobarbital [37], authors identified published pharmacokinetic models (7–8 per study) in the literature with differences in populations supplying the data, model structure, and model purpose. Each of these studies was focused on application of prior popPK models in the context of therapeutic drug monitoring in patient populations or contexts different than the originating models. It was noted that while most of the published models evaluated in these studies had evaluated goodness of fit by standard techniques (visual predictive checks, bootstrap re-estimation of parameters to establish confidence intervals), very few performed more extensive validation using a withheld data set. Predictive performance of the models was evaluated by calculation of prediction error and several derivatives thereof (mean error, mean absolute error, and root mean-square error).

One shortcoming of both statistical Learning techniques, such as tree-based methods, and pharmacometric (NLME) modelling is that they are both limited to a particular representation of the data. A tree-based heart failure model that makes predictions based on age, gender and lifestyle factors or an NLME model that makes pharmacokinetic predictions based on body size, gender and renal functions is limited by how these predictors are represented. If we were to give the heart failure model the MRI scan of a heart, for example, it would not be able to use this data for predictions because there is little connection between individual pixels from an MRI image and heart failure. Solving this problem, known as the representation problem [38], would enable researchers to validate their model over a wider range of data, leading to more robust models. Deep learning solves this problem by determining both the representation of the data and the mapping of data to the output. It does this by discovering representations that are expressed in other simpler representations. For example, deep learning methods used in image recognition, might detect simpler aspects of the image such as edges in one layer of the neural network, corners and contours in another layer until the object is identified. To extend a tree-based model for heart failure so that it also can use MRI images, for example, one could generalize techniques that combine metadata with image classification [39]. These techniques leverage convolutional neural networks (CNNs) for the images with relatively small feed forward networks for the metadata. A feedforward is a common neural network architecture in which information flows from the input to the output without any feedback connections while a convolutional neural network is a particular type of feed forward network designed for image processing [38]. By taking the output from the feedforward network and merging it with the CNN’s feature vector, we can create a single neural network. For the heart failure application envisioned here, we could use a feed forward network to replace the tree-based heart failure model and combine that with a CNN for the MRI images.

Finally, it is important to realize that NLME modeling and Statistical/Deep Learning are active areas of research which could lead to new insights into how to validate these models and even what it *means* to validate these models. A good example is the empirical study by Zhang et al. [40]. They show empirically that that many successful neural network architectures are complex enough to memorize the training data. Yet, despite this apparent overfitting, deep neural networks are remarkably accurate on test data, even if explicit regularization is not used. While there is no universally accepted explanation, a consensus is beginning to emerge from empirical studies based on a notion that Belkin defines as the capacity of a function [41]. Roughly speaking, the capacity of a function is the number of parameters needed to specify a particular class of functions. When the capacity of a function is relatively low, as typically happens in classical statistical learning, the function will exhibit the bias-variance tradeoff: the function will be complex enough to capture the underlying structure in the test data without fitting to noise in the training data. However, as the capacity of the function increases, say, by increasing the number of hidden layers in a neural network, the function can fit the training data perfectly without exhibiting poor performance on the test data, thereby avoiding the danger of over fitting the model. Belkin explains this behavior by showing that the performance of many neural networks is described by a double decent risk curve rather than the classic U-shaped curve. When the capacity of the function increases above the interpolation threshold, the double decent risk curve shows that the model typically performs better on test data than the classical U-shaped risk curve would predict. From this empirical observation, Belkin argues that the capacity of a neural network is not a good measure of a function’s ability to generalize beyond the training data. Belkin’s observation is consistent with the Universal Approximation Theorem [40], which states that a neural network can approximate any function sufficiently close given enough hidden layers and a deterministic relation between the input variables and the target function.

### Implications for QSP modeling

Combining statistical and deep learning model with QSP holds much promise. For example, researchers have pointed out that findings from statistical learning models, such as importance of left ventricular ejection fraction for predicting survival in patients with heart failure, can be incorporated into QSP models [41]. In addition, recent work [42] suggests that neural ordinary differential equations, which specify the time derivatives of state variables by a neural network, could lead to mechanistic models that illuminate the underlying biologic processes that affect pharmacokinetics and dynamics of drugs.

While the concepts of internal and external validation are certainly applicable to the QSP context, they do not fully cover the conceptual validation process described herein. In a QSP model, we strive to demonstrate that we have adequately captured the correct biology, and that model trajectories are generated for the correct reasons. Thus, we focus on a pathway-level view of validation; this will always be an external validation in the statistical sense, but not all external validation data sets will provide comparable levels of biological validation. We here have introduced the concepts of qualification, within-target, within-pathway, and cross-pathway validation to describe the levels of biological validation we can pursue. Note here that the latter three of these biological levels would properly be considered an external validation, as each represents experimental data not used during calibration; however, the magnitude of information each test provides upon success (or failure) is not conceptually equal.

Once last implication for validation in QSP analyses is the question of what happens if a model (or VPop) fails validation. In most statistical contexts, a model that fails validation on a data set it should predict would be rejected and the model building process begun anew. The failure could happen for many reasons (failure of the data to conform to the assumptions of the model, inadequate amount or quality of training data, overfitting, etc.), but regardless of the cause, a fundamentally different model must be considered instead. However, as discussed in Hendriks 2013, the purpose of the QSP model is to facilitate understanding of the key biology and pharmacology at play [43]. Thus, a failure in validation should help drive the future development of the model and itself provide key insights into how the pathways interact between each other and with various interventions. The reasons for failure of a QSP model to validate are varied (insufficient biological representation, parameters estimated from one context that do not translate to another, inability of the calibration data to properly constrain components of a VPop, among many other possibilities), but failure of the model to validate followed by careful examination of why this occurred can be a critical learning point along the path of model development. An example of this is shown in Hamuro et al. [44]. The authors applied the model from [27, 45] to a novel context, finding the model accurately predicted one context but not another. However, after consulting the literature and adding new biology to the model (without needing to recalibrate any parameters from the original model), they found the updated model could now predict both the original data as well as the new data.

## Conclusions

In this review, we have discussed the concept of validation of QSP models, how validation approaches are presented in the QSP literature, and how those approaches both draw from and differ from validation approaches in the statistical and pharmacometric fields. We presented a heuristic for QSP model validation that balances validation of both the knowledge and the data used to build the model, by considering how different components of a model interact with one another. This heuristic is inspired by the multiple levels of data (biochemical, cellular, tissue, organism, population) from which a QSP model is constructed and subsequently applied. By validating in not only the statistical sense (e.g. assessing quality of parameter estimates) but also in the biological sense the representation contained within the model, the QSP modeler is more able to generate confidence in the mechanistic hypotheses proposed by the modeling analysis. Of course, not every analysis will have a dataset capable of powering all levels of validation discussed here. However, applying the approach of within-target, within-pathway, and cross-pathway validation can also assist the modeler to propose feasible new investigations which can either begin to demonstrate validity of the model, or uncover new biology necessary for the model to properly describe the pathways under study.
